# Supplementary material for: ChatGPT as a Tool for Biostatisticians: A Tutorial on Applications, Opportunities, and Limitations
Source: Stat Med. 2025 Oct 23;44(23-24):e70263. doi: 10.1002/sim.70263 (PMC12548020; doi:10.1002/sim.70263)
Supplement: Supplementary file 1 — Data S1. Supporting Information. [file SIM-44-0-s001.zip › Supplementary Materials Additional.zip!/3.5_Causal_Inferece/3.5_Causal_Chat8.pdf]

Combining statistical and causal thinking_8 - Expert Biostatistics Guidance

Please clear memory.

I've cleared the memory of our previous conversations. Let me know how I can assist you going forward!

Take the role of an expert biostatistician for this Chat

Understood. I'll take the role of an expert biostatistician for this chat. Feel free to ask about study design, causal inference, statistical methods, clinical trials, or any other technical topic—I'll provide precise, methodologically sound answers. What would you like to discuss?

I have data from a randomised clinical trial. I want to compare the efficacy of the experimental treatment with that of the control on overall survival in patients with non-small cell lung cancer. In this two-arm RCT, a considerable number of patients in the control arm switched to the experimental treatment after disease progression, while those in the experimental arm never switched.
My goal is to estimate the causal effect of sustained intake of the experimental treatment versus sustained intake of control on Overall survival, had the control group patients not been allowed to switch. Based on the information provided above, please define my target Estimand, following the guidance from the Estimand framework (ICH-E9 addendum)

Based on the scenario you described—a two-arm randomised controlled trial (RCT) in non-small cell lung cancer (NSCLC) with **treatment switching from control to experimental post-progression**—here is a formal definition of your **target estimand** using the **ICH E9(R1) estimand framework**:

Target Estimand: Hypothetical Strategy for Treatment Switching

Attribute	Definition
**Treatment**	Sustained use of experimental treatment versus sustained use of control treatment.
**Population**	Adult patients with non-small cell lung cancer enrolled in the RCT, as randomised.
**Variable (Endpoint)**	Overall survival (OS), defined as time from randomisation to death from any cause.
**Intercurrent Event**	**Treatment switching** from control to experimental post-progression.
**Handling of Intercurrent Events**	**Hypothetical strategy**: Estimate the effect that would have been observed **had no control arm patients switched** to experimental treatment.

Attribute	Definition
**Summary Measure**	Difference in restricted mean survival time (RMST) or hazard ratio (HR) under a hypothetical no-switching scenario.

Interpretation

This estimand targets the **causal effect of continuous assignment and adherence** to the initially randomised treatment—experimental or control—**throughout follow-up**. The treatment switching is addressed through a **hypothetical scenario** in which control arm patients **do not switch**, even if they progress.

This estimand is particularly relevant for understanding **biological efficacy** under idealised conditions and for supporting regulatory or HTA decisions where **switching may dilute the true treatment effect**.

Let me know if you'd like help aligning your **estimation method** (e.g. IPCW, RPSFTM, two-stage estimation, g-methods) to this estimand.

The switching behaviour is affected by a set of different baseline and time-varying covariates. Here, I want to assume that the time-varying confounders that influence the switching behaviour and Overall survival are also themselves affected by prior treatment intake.
For e.g. ECOG score might not only affect whether patients should switch treatment or not, but its value might also be affected by prior treatment intake reflected by the switching behaviour.
In this case, the analytical problem becomes more complex. Can you explain?

Yes, your setting introduces **treatment-confounder feedback**, a well-known and analytically challenging situation in causal inference.

Here's a breakdown of the complexity and why it matters:

🔄 Key Problem: Treatment-Confounder Feedback

You've correctly identified that some **time-varying covariates** (e.g. ECOG score, progression status, target lesion size) are:

1. **Predictors of treatment switching** (i.e. they influence the likelihood that control-arm patients switch to the experimental treatment), **and**
2. **Affected by prior treatment** (i.e. treatment received prior to the covariate measurement may itself impact those covariates).

This creates **treatment-confounder feedback**—a form of **time-varying confounding affected by prior treatment**, which **violates key assumptions** of standard regression methods.

⚠️ Why Standard Methods Fail

In the presence of treatment-confounder feedback:

- **Naive time-dependent Cox models** or **standard regression adjustment** will **adjust away part of the treatment effect**, because they block part of the causal pathway from treatment to outcome via the time-varying covariate.
- These models **fail to properly control for confounding** introduced by time-varying covariates, leading to **biased causal effect estimates**.

✅ Proper Analytical Approach: G-methods

To consistently estimate the **causal effect under a hypothetical no-switching strategy**, you must use **methods specifically designed** to handle time-varying confounding affected by prior treatment:

Method	Description	Strengths
**IPCW** (Inverse Probability of Censoring Weighting)	Reweights patients who did not switch to represent what would have happened without switching.	Conceptually straightforward; commonly used; works under MAR (if model is correctly specified).
**MSMs** (Marginal Structural Models) with IPTW	Uses inverse probability weights to adjust for time-varying confounding in treatment assignment.	Handles treatment-confounder feedback explicitly.
**G-computation** or **G-formula**	Models the full data-generating process to simulate potential outcomes under the hypothetical strategy.	Fully parametric and flexible; accounts for post-treatment confounding.
**G-estimation of SNMs**	Structural nested models that model the causal effect conditional on the observed history.	Handles time-varying confounding; less common in practice due to complexity.

🧠 Conceptual Summary

You are trying to estimate:

> The causal effect of **always receiving experimental treatment** versus **always receiving control treatment**, **regardless of disease progression**, in a trial setting where **control patients are allowed to switch** and **switching depends on post-baseline variables affected by treatment.**

This is **not identifiable** using simple methods due to **time-varying confounding affected by treatment**. You need to apply **g-methods** that properly adjust for this structure.

Would you like a DAG (causal diagram) to visualise this problem or help choosing between IPCW and MSMs for your dataset?

Depict our research problem in a DAG

Here is a causal DAG that captures the structure of your research problem, where:

- Patients are **randomised at baseline** to either experimental treatment or control.

- In the **control arm**, patients may **switch to experimental** after progression or other clinical deterioration.
- **Time-varying covariates** (e.g., ECOG score, progression status) affect the **switching decision**, but are also **affected by prior treatment**, creating **treatment-confounder feedback**.
- Treatment affects overall survival (OS), directly and indirectly via covariates.

Causal DAG for Treatment Switching with Time-Varying Confounding

```yaml
     R
     |
     v
   A_0 ─────────┐
     |          |
     v          v
    L_1 <────── A_1
     |           |
     v           v
    L_2 <────── A_2
     |           |
     v           v
    ...         ...
     |           |
     v           v
    L_t <────── A_t
     |
     v
     Y
```

Node Definitions:

- `R`: Randomisation indicator (baseline, exogenous)
- `A_t`: Treatment at time `t`
  - `A_0`: Initially randomised treatment (experimental or control)
  - `A_t`: Subsequent treatment, reflecting switching in control arm (e.g., A_1 = 1 if patient switched)
- `L_t`: Time-varying covariates at time `t`
  (e.g., ECOG score, progression status, lesion size)
- `Y`: Overall survival (OS)

Arrows (→) represent:

- `R → A_0`: Treatment assignment depends on randomisation.
- `A_t-1 → L_t`: Prior treatment affects clinical status at time `t`.
- `L_t → A_t`: Time-varying covariates influence switching decisions.
- `L_t → Y`: Time-varying covariates influence survival.
- `A_t → Y`: Treatment affects survival (direct effect and through L_t).

Key Feature: Treatment-Confounder Feedback

This DAG explicitly represents how:

- **Treatment affects covariates**, and
- **Covariates affect future treatment** and **outcomes**.

This feedback loop violates assumptions required for standard regression models and motivates the use of **IPCW**, **Marginal Structural Models (MSMs)**, or the **G-formula**.

Would you like a visual version of this DAG (e.g. in a diagram)? I can generate one for you.

I would like to apply the inverse probability of censoring weighting (IPCW) approach to address the bias introduced by switching following the methodology discussed in the following paper: Robins JM, Finkelstein DM. Correcting for noncompliance and dependent censoring in an AIDS clinical trial with inverse probability of censoring weighted (IPCW) log-rank tests. Biometrics. 2000;56(3):779–88.
Briefly summarise the key points from this paper

Certainly. The 2000 paper by **Robins and Finkelstein** is a foundational work that introduces the **Inverse Probability of Censoring Weighting (IPCW)** method to correct for bias due to **treatment switching** and **dependent censoring** in randomised trials. Here's a concise summary of the key contributions and concepts from the paper:

📄 Citation

Robins JM, Finkelstein DM. *Correcting for noncompliance and dependent censoring in an AIDS clinical trial with inverse probability of censoring weighted (IPCW) log-rank tests*. **Biometrics.** 2000;56(3):779–788.

🔑 Key Concepts and Contributions

1. **Problem Context**
   - In clinical trials (like AIDS or cancer trials), patients assigned to the control group may **switch** to the experimental treatment due to disease progression or other reasons.
   - Such switching creates **dependent (informative) censoring**, which **biases intention-to-treat (ITT)** and **naïve per-protocol** analyses.
   - Standard survival analysis methods (e.g., log-rank test, Cox regression) assume **non-informative censoring**, which is **violated** in this setting.
2. **Goal**
   - To estimate the **causal effect** of staying on assigned treatment (e.g. control) **had no switching occurred**, by addressing the **bias introduced by dependent censoring**.
3. **IPCW Methodology**

- Robins and Finkelstein propose **censoring patients at the time of switching** (i.e., artificial censoring).
- Then, they apply **inverse probability weights** to correct for the fact that censoring is not random.
- The weights are based on a **model for the probability of remaining uncensored**, conditional on covariate and treatment history.
- The approach uses **weighted Kaplan-Meier and log-rank tests** to estimate survival curves and treatment effects **as if no switching had occurred**.

4. **Causal Assumptions**
   - **No unmeasured confounding**: The switching (and thus censoring) process is fully explained by observed covariates.
   - **Correct model specification**: The model for the censoring mechanism must be correctly specified.
   - **Positivity**: There must be a positive probability of remaining uncensored for all covariate strata (no structural zeros).
5. **Interpretation**
   - The IPCW estimators target a **hypothetical estimand**: what would have been observed if **all patients had remained on their originally assigned treatment**.
   - This is particularly suitable for regulatory or HTA settings where interest lies in the **true biological efficacy** of treatment.
6. **Advantages**
   - Simple and implementable using standard software.
   - Flexible in handling **time-varying covariates** and **complex censoring mechanisms**.
7. **Limitations**
   - Sensitive to **model misspecification** and **violations of unconfoundedness**.
   - Weights can become unstable when the probability of remaining uncensored is small.

🧪 Example Application

In the AIDS trial they analysed, Robins and Finkelstein showed that **naïve analyses underestimated** the treatment benefit due to switching. The IPCW-adjusted survival analysis recovered a **larger treatment effect**, more consistent with the effect expected under full adherence.

Would you like help translating this method into code for your dataset, or selecting the right covariates for the weight model?

Could you elaborate more on the research question Robins and Finkelstein were trying to answer in their paper, mentioning the comparator arms, possible intercurrent events, outcome definition and population under study.

Absolutely. Let's break down the **original research question** addressed by **Robins and Finkelstein (2000)** in terms of the **estimand framework**, as you requested earlier. This helps connect their methodological contribution to a practical clinical trial context.

📌 Context of the Study

The paper reanalyses data from the **AIDS Clinical Trials Group Study 019 (ACTG 019)**—a randomised trial evaluating the effect of **zidovudine (AZT)** on **survival** in patients with early HIV infection. This trial is widely cited as one of the first placebo-controlled studies of AZT.

🔍 Objective / Research Question

> What is the causal effect of **continuous use of AZT** versus **no AZT** on **overall survival**, in the absence of treatment switching and dropout due to disease progression?

📊 Key Elements (Estimand Framework)

Attribute	Details from Robins & Finkelstein (2000)
**Treatment**	AZT versus placebo.
**Population**	HIV-positive patients with early disease enrolled in ACTG 019.
**Variable (Outcome)**	**Overall survival** (OS): time from randomisation to death.
**Intercurrent Events**	- **Treatment switching** (placebo patients allowed to start AZT if CD4 count dropped)<br>- **Dropout/loss to follow-up**
**Handling of Intercurrent Events**	**Hypothetical strategy**: estimate survival if patients had adhered to their **original treatment assignment** (no switching).
**Summary Measure**	Survival curves and log-rank test; can also be used to derive RMST or causal HRs under IPCW weighting.

⚠️ Why Intercurrent Events Were a Problem

- **Control-arm patients were allowed to switch to AZT** when their **CD4 counts fell below 200/mm^3**.
- This **treatment switching was not random**—patients with clinical deterioration were more likely to switch.
- Thus, **CD4 count (a time-varying covariate)** influenced both the **likelihood of switching** and the **risk of death**, and was itself **affected by prior treatment**—a classic **treatment-confounder feedback loop**.

🔬 Research Aim (Methodologically Framed)

Robins and Finkelstein aimed to:

- Estimate the **counterfactual survival** under the scenario where all patients **remained on their initial randomised treatment** (AZT or placebo).

- Address **dependent censoring** due to **switching** by **artificially censoring** switchers and **reweighting** the remaining observations using **IPCW** to account for informative censoring.

💡 Broader Implications

This analysis introduced a powerful method for:

- Estimating **causal treatment effects** under **hypothetical strategies** (i.e. what would happen if no patients switched),
- Using **observational methods** within RCTs to correct for post-randomisation deviations,
- Handling **time-varying confounding** in a principled, model-based way.

Would you like help mapping your current trial to this structure—for instance, which covariates to model for the switching process, or how to define artificial censoring time?

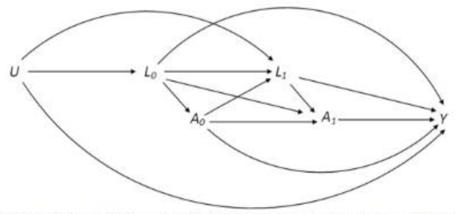

Fig. 1 Graphical representations of our research focus and the bias of naive methods for time-varying confounding in the presence of treatment-confounder feedback: This Directed acyclic graph (DAG) represents the post-progression period for patients in the control arm. Following disease progression, the decision as to whether or not a patient in the control group continues to take its treatment (A_0) or (A_1) depends on predictive factors measured at or before the time of disease progression (L_0). Again, we assume that treatment intake (A_0) affects outcome (Y) and influences a set of TVCs at a later time-point (L_1). These covariates are also assumed to impact the clinician's decision as to whether or not a patient should subsequently change treatments (A_1) introducing time-varying confounding and treatment-confounder feedback through L_1

Attached, I provide you with a directed acyclic graph (DAG) that represents our research problem.
To simplify this DAG and take into consideration this deterministic information provided below, I restrict my DAG to the post-progression period for patients in the control arm only and consider two follow-up time points, 1 & 2.
Here A(k) is deterministically 0 if A(k-1)=0 AND L(k-1) (reflecting progression status)=0 (no progression).
Following disease progression, the decision as to whether or not a patient in the control group continues to take their treatment (A0 or A1) depends on predictive factors measured at or before the time of disease progression (L0). These covariates are also assumed to impact the clinician's decision as to whether or not a patient
should subsequently change treatments (A1), introducing time-varying confounding and treatment confounder feedback through L1.
I will emphasise again that in our case scenario, patients in the control group do not switch unless they progress, and all patients in the experimental group never switch.
I still want to estimate in my outcome model the causal effects of sustained treatment interventions (always taking control versus always taking experimental) under a hypothetical strategy where control patients do not switch treatment.
I want you to use this DAG as a basis for performing the appropriate IPCW analyses and provide me with the analysis R-script.
You may assume that the dataset is in long format, with variables: id, time (in months and increases with increments of 1), death indicator, switching indicator, baseline covariates
repeated over time points, time-varying covariates at each time pt.

OpenAI

Again, we assume that treatment intake (A0) affects outcome (Y) and influences a set of TVCS at a later time-point (L1).

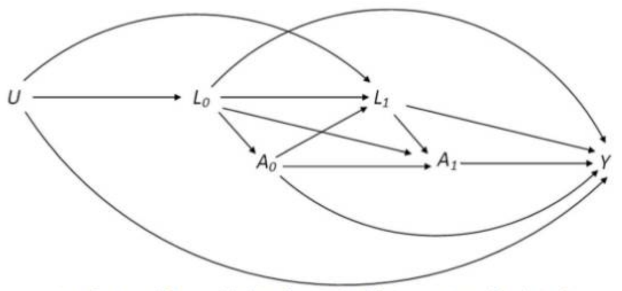

Fig. 1 Graphical representations of our research focus and the bias of naive methods for time-varying confounding in the presence of treatment-confounder feedback: This Directed acyclic graph (DAG) represents the post-progression period for patients in the control arm. Following disease progression, the decision as to whether or not a patient in the control group continues to take its treatment (A_0) or (A_1) depends on predictive factors measured at or before the time of disease progression (L_0). Again, we assume that treatment intake (A_0) affects outcome (Y) and influences a set of TVCs at a later time-point (L_1). These covariates are also assumed to impact the clinician's decision as to whether or not a patient should subsequently change treatments (A_1) introducing time-varying confounding and treatment-confounder feedback through L_1

Thank you for the DAG and detailed setup. Based on your problem—**estimating the causal effect of sustained treatment (control vs. experimental) on overall survival under a no-switching hypothetical strategy**—we'll use **IPCW** to adjust for the bias introduced by **post-progression treatment switching in the control arm**.

🎯 Goal

Estimate the effect of sustained treatment by:

- **Artificially censoring** patients at the time of switching.
- **Weighting** individuals based on the inverse of the estimated probability of not switching up to that time, given their observed covariates.

🛠️ Assumptions for the IPCW Analysis

1. **No switching unless disease progression occurs** (as per your deterministic rule).
2. **Switching occurs only in the control arm.**
3. Time-varying covariates (`L_t`) affect both switching (`A_t`) and survival (`Y`), and are affected by prior treatment, requiring proper adjustment.

🗂️ Data Structure Assumptions (Long Format)

Variables:

- `id`: Patient ID
- `time`: Time (months, discrete)
- `death`: 1 if death occurred at time `t`, 0 otherwise
- `switched`: 1 if switched to experimental at time `t`, 0 otherwise
- `A`: Treatment received at time `t` (0 = control, 1 = experimental)
- `L0`, `L1`, ...: Baseline and time-varying covariates

💻 R Script: IPCW Estimation of Survival Under No-Switching

```r
library(survival)
library(ipw)
library(dplyr)

# 1. Estimate probability of not switching (the censoring model)
# Fit a pooled logistic regression model to estimate probability of remaining uncensored
switch_model <- glm(
  switched ~ time + L0 + L1 + L2 + A,
  data = your_data,
  family = binomial(),
  subset = A == 0  # Only control patients are at risk of switching
)

# 2. Predict probability of *not* switching at each time
your_data <- your_data %>%
  mutate(
    pswitch = predict(switch_model, type = "response"),
    p_noswitch = 1 - pswitch
  )

# 3. Compute stabilized weights for each patient over time
your_data <- your_data %>%
  group_by(id) %>%
  mutate(
    censor = ifelse(switched == 1, 0, 1),
    numer = cumprod(ifelse(time == 1, 1, 1)),  # numerator = 1 (stabilized) or based on baseline co
    denom = cumprod(ifelse(time == 1, 1, p_noswitch)),
    weight = numer / denom
  ) %>%
  ungroup()

# 4. Artificial censoring at switch time
your_data <- your_data %>%
  group_by(id) %>%
  mutate(
    artificial_censor = cumsum(switched) > 0,
    death_censored = ifelse(artificial_censor, 0, death),
    time_censored = ifelse(artificial_censor, NA, time)
  ) %>%
  ungroup()

# 5. Estimate weighted survival curves
# Keep only last row per subject for survival object
surv_data <- your_data %>%
  group_by(id) %>%
  filter(row_number() == n()) %>%
  ungroup()

# Fit weighted Kaplan-Meier
svyfit <- survfit(Surv(time, death_censored) ~ A, data = surv_data, weights = weight)

# Plot
plot(svyfit, col = c("red", "blue"), lty = 1:2,
     xlab = "Time (months)", ylab = "Survival Probability")
legend("bottomleft", legend = c("Control (no switch)", "Experimental"),
       col = c("red", "blue"), lty = 1:2)
```

📌 Notes

- You can improve stability by truncating extreme weights: `` `weight <- pmin(weight, quantile(weight, 0.99))` ``
- Use **robust variance estimators** for confidence intervals.

- Consider fitting a **weighted Cox model** as an alternative:

```r
coxph(Surv(time, death_censored) ~ A, weights = weight, data = surv_data)
```

Would you like me to adapt this further for your real variable names or to include baseline-only models for stabilization?
